# Unexplained Groin Pain in the Elderly: Spontaneous Iliopsoas Tendon Rupture With Haematoma in a 90-Year-Old Woman

**DOI:** 10.7759/cureus.94601

**Published:** 2025-10-14

**Authors:** Praveenraja Shanmugam, Sona Murugan, Felix Saka

**Affiliations:** 1 General Medicine, Peterborough City Hospital, North West Anglia NHS Foundation Trust, Peterborough, GBR; 2 General Internal Medicine, Doncaster and Bassetlaw Teaching Hospitals NHS Foundation Trust, Doncaster, GBR; 3 Geriatrics, Peterborough City Hospital, North West Anglia NHS Foundation Trust, Peterborough, GBR

**Keywords:** atraumatic hip pain, conservative management, elderly female, iliopsoas haematoma, iliopsoas tendon rupture, spontaneous tendon injury, warfarin therapy

## Abstract

Hip and groin pain are frequent presenting complaints among elderly patients. These symptoms are most commonly related to fractures, which are usually identifiable on X-rays. Spontaneous iliopsoas tendon injury, however, is a rare and under-recognised cause of atraumatic hip or groin pain in this population. We report the case of a 90-year-old woman with a history of multiple comorbidities on long-term warfarin for atrial fibrillation who presented with dull left groin pain radiating to the lower back and marked limitation of mobility in the absence of recent trauma. On admission, the international normalised ratio (INR) was 3.98 (reference range, 2-3) and warfarin was held; haemoglobin (Hb) was 87 g/L (reference range, 115-165 g/L), which is the patient's baseline with no fall on serial checks. Examination revealed severe pain on resisted hip movement and groin tenderness, while pelvic X-rays excluded fracture. CT imaging demonstrated bulky soft tissue tracking along the left iliacus muscle extending to the lesser trochanter, consistent with spontaneous iliopsoas tendon rupture with intramuscular haematoma. Orthopaedics recommended conservative management with analgesia, mobilisation as tolerated, and inpatient rehabilitation. MRI was not pursued as it would not have altered the management. The patient remained haemodynamically stable without any drop in haemoglobin levels. This case highlights the importance of considering spontaneous iliopsoas tendon rupture in elderly patients who present with atraumatic hip or groin pain and have non-diagnostic X-rays. Timely cross-sectional imaging is often key to making the diagnosis. Early diagnosis allows appropriate conservative management and is associated with favourable functional outcomes.

## Introduction

Hip and groin pain are common presenting complaints among elderly patients, with hip pain affecting approximately 14% of adults aged ≥60 years [1]. Specific prevalence data for groin pain are limited, but it is often clinically significant in this population. The usual presentation is due to a fall and fracture (e.g., neck of femur or pelvic fracture), which can be readily identified on X-rays. Spontaneous iliopsoas tendon rupture (ITR) is more common among athletes but is very rarely reported in older people, with a prevalence of about 0.66% [2-4]. 

The iliopsoas muscle complex, comprising the psoas major and iliacus muscles, is the primary hip flexor. It originates from the lumbar vertebrae and iliac fossa, inserting onto the lesser trochanter of the femur. The iliopsoas also contributes to hip flexion, adduction, and external rotation, and assists in trunk flexion when the lower limbs are fixed [5]. Risk factors for spontaneous ITR include advanced age, female sex, chronic steroid use, fluoroquinolone exposure, osteopenia, malignancy, and systemic inflammatory diseases [2,4,6,7]. Coagulopathy and antithrombotic therapy have also been reported with rupture-related haematoma [2,3].

MRI is the investigation of choice, but CT is often used in acute care and can demonstrate diagnostic features [2,4,6,8]. We report the case of a 90-year-old woman who presented with groin and hip pain and was diagnosed with the rare entity of spontaneous iliopsoas tendon rupture. She was on long-term warfarin for atrial fibrillation and had multiple comorbidities. Conservative management, including careful monitoring of anticoagulation and structured rehabilitation, led to a favourable functional outcome. This case highlights the importance of early recognition and timely imaging in elderly patients with atraumatic hip or groin pain.

## Case presentation

A 90-year-old woman presented with dull, left groin pain radiating to the left lower back. Before this episode, she could mobilise independently with a frame, but her mobility had become significantly limited. She reported a fall six months prior to this admission but denied any other recent fall or trauma. Her past medical history included congestive cardiac failure, vertebral fracture, osteoarthritis, peripheral neuropathy, two episodes of transient ischaemic attack, visual impairment, diverticular disease, hypertension, and mild cognitive impairment. She was on long-term warfarin for atrial fibrillation.

On examination, there was severe pain on hip flexion and extension against resistance. She could not perform a straight leg raise due to pain. On palpation, there was tenderness over the left groin and lower back without visible swelling, and sensation was intact. A pelvic X-ray ruled out fracture and showed mild bilateral osteoarthritic changes in the hip joints with vascular calcifications. Admission bloods showed international normalised ratio (INR) 3.98 (reference range, 2-3) and warfarin was held. Haemoglobin was 87 g/L (reference range, 115-165 g/L), which was her baseline with no subsequent drop. 

**Figure 1:**
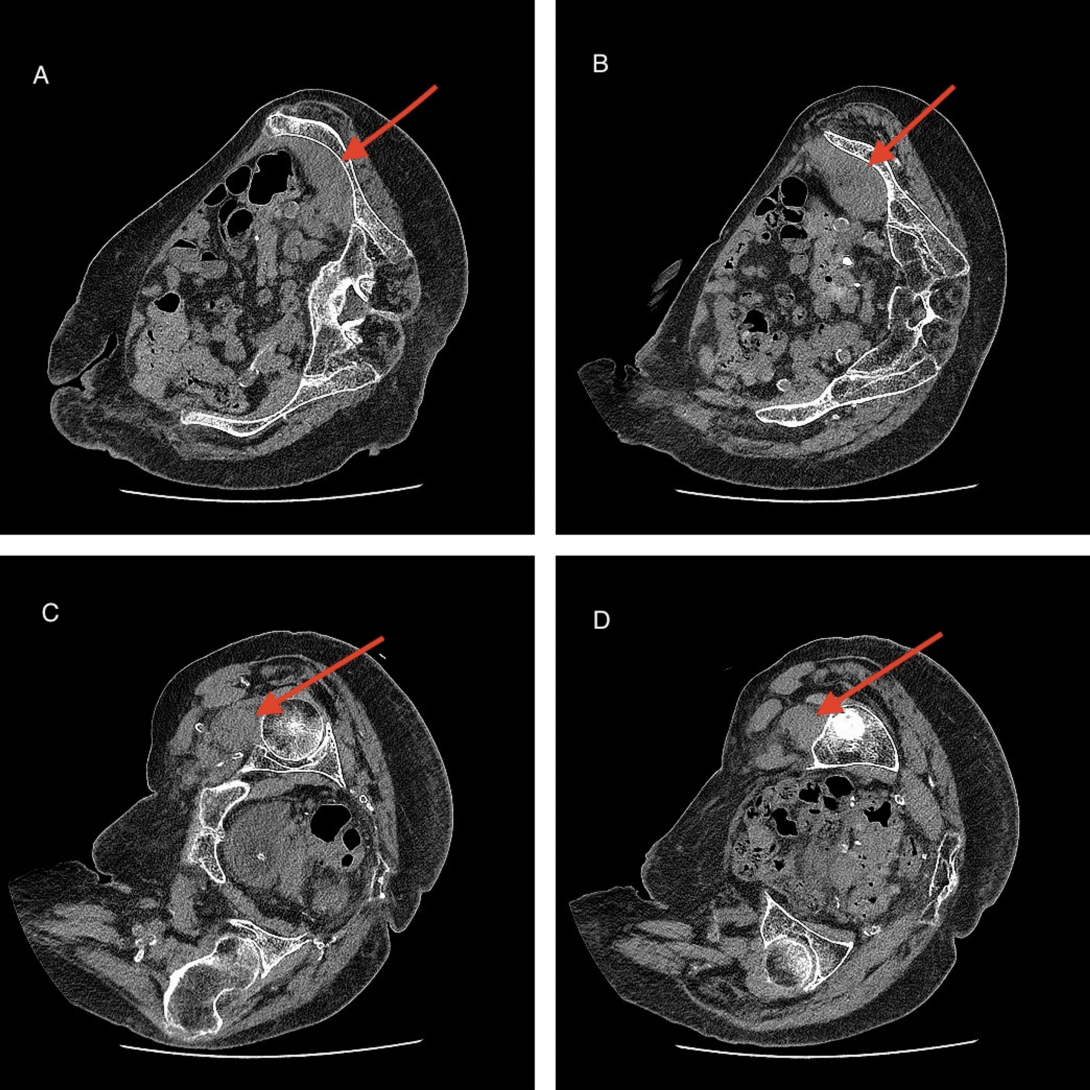
CT pelvis showing spontaneous iliopsoas tendon rupture (A) Axial CT at the level of the iliac crest showing bulky soft tissue along the left iliacus muscle (arrow). (B) Axial CT at the mid-pelvis demonstrating extension of the abnormal soft tissue along the iliacus muscle (arrow). (C) Axial CT at the hip joint level showing tracking of the soft tissue towards the lesser trochanter (arrow). (D) Axial CT at the subtrochanteric level confirming bulky soft tissue consistent with iliopsoas tendon rupture (arrow).

CT pelvis showed no acute proximal femoral, pubic ramus, or sacral fracture, but revealed bulky soft tissue along the left iliacus extending to the lesser trochanter, consistent with an iliopsoas muscle-tendon rupture with haematoma (Figure 1). CT was selected for its rapid availability and practicality in this elderly patient, providing sufficient information to guide management.

The orthopaedic team confirmed a diagnosis of iliopsoas tendon rupture with haematoma. Given the patient’s haemodynamic stability, conservative management was recommended. Warfarin, temporarily withheld on admission, was safely restarted at a low dose once therapeutic anticoagulation was achieved, without the need for reversal therapy. Analgesia was optimised in a stepwise manner, starting with regular paracetamol and escalating to codeine as required, with additional PRN (*pro re nata*; as needed) doses for breakthrough pain. Pain was monitored clinically, guiding the progression of activity.

The physiotherapy team assessed the patient following the orthopaedic review. A structured mobilisation plan was implemented, beginning with transfers from bed to chair, progressing to assisted standing with a walking frame, and eventually walking short distances as tolerated. Weight-bearing was allowed as pain permitted, with gradual increases under supervision.

After seven days of hospitalisation and clinical improvement, the patient was discharged to an inpatient rehabilitation facility for continued physiotherapy and functional recovery. By discharge, the patient was able to perform independent bed-to-chair transfers and ambulate short distances with a walking frame with minimal assistance. Pain control had improved sufficiently to allow active participation in physiotherapy.

## Discussion

Spontaneous iliopsoas tendon rupture is rare, with an estimated prevalence of approximately 0.66% in cohorts including both traumatic and atraumatic injuries [2-4,6]. It predominantly affects older women, and our patient falls within this demographic. Prior reports have linked this condition to chronic corticosteroid or fluoroquinolone use, as well as systemic inflammatory disease; however, none of these factors were present in our patient. Therefore, age-related degeneration is the most likely underlying mechanism [2,4,6,7].

Typical features include acute, atraumatic groin or hip pain, impaired mobility, and pain-limited hip flexion, all of which were observed in our patient [4,6]. A palpable mass from tendon retraction has been described in some cases, such as by Ramanan et al. [7]; however, this finding was not appreciated in our patient, consistent with the heterogeneity of examination findings.

The differential diagnosis for atraumatic hip or groin pain in elderly patients is broad and includes both intra-articular and extra-articular causes. Intra-articular causes such as osteoarthritis, femoroacetabular impingement, or acetabular labral tears may present with anterior hip pain, while extra-articular sources include iliopsoas tendinopathy, rectus femoris strain, adductor muscle injury, and trochanteric bursitis. Referred pain from lumbar spine pathology or intra-abdominal and intrapelvic processes, including hernias or vascular lesions, should also be considered. Careful clinical assessment and cross-sectional imaging are essential to distinguish spontaneous iliopsoas tendon rupture from these more common conditions [9].

X-rays are frequently non-diagnostic in iliopsoas tendon injury, as in our case. CT can reveal soft-tissue swelling/haematoma along the iliopsoas and is often the practical first-line cross-sectional modality in emergency pathways. In our patient, CT showing bulky soft tissue tracking to the lesser trochanter was characteristic [2,4,6,8]. MRI remains the gold-standard investigation. However, management is commonly conservative when CT findings and clinical assessment are concordant, and the patient is clinically stable. MRI may be reserved for cases with diagnostic uncertainty or suspected complications [2-4,6,8]. Distal involvement near the lesser trochanter, with greater susceptibility of the medial psoas tendinous component and relative preservation of lateral iliacus fibres, has been described and accords with this distribution [7].

Haematoma related to iliopsoas tendon rupture has been reported in patients on anticoagulation or with coagulopathy [2,3]. Our patient was on long-term warfarin and presented with an INR of 3.98 (reference range, 2-3). CT pelvis showed iliopsoas rupture with haematoma, but as she was haemodynamically stable and her haemoglobin remained at baseline without any drop, the decision was to hold warfarin and continue with conservative management.

## Conclusions

We strongly emphasise early recognition of spontaneous iliopsoas tendon injury in elderly patients presenting with atraumatic acute hip or groin pain when X-rays are non-diagnostic, because timely cross-sectional imaging prevents delays and excludes important mimics such as occult fracture and malignancy, and can also identify associated intramuscular haematoma, particularly in anticoagulated patients. In acute settings, CT is often sufficient to establish the diagnosis, with MRI reserved for uncertainty or suspected complications.

Conservative management with structured rehabilitation generally yields good functional outcomes, while invasive therapy is reserved for haemodynamic instability, expanding haematoma, or compressive features. In patients on anticoagulation, antithrombotic therapy should be reviewed individually, and careful monitoring for haematoma progression is essential. We acknowledge that this report reflects the experience of a single patient, without MRI confirmation and long-term follow-up; nevertheless, it provides practical insights into the presentation, diagnosis, and management of this rare condition.
